# Dataset of 12,161 steel rebar tests from sudanese construction projects (2016-2022)

**DOI:** 10.1016/j.dib.2026.112469

**Published:** 2026-01-15

**Authors:** Amged O. Abdelatif, Abdelrahim H. Abdelrahim, Gamar-Aldwla S. Shangray, Mohammed-Alfatih Mustafa, Mustafa M. Abaker, Yahia A. Idris, Abdelrahim M. Yousif

**Affiliations:** Department of Civil Engineering, Faculty of Engineering, University of Khartoum, Khartoum, P. O. Box 321, 5111, Sudan

**Keywords:** Steel reinforcement, Tensile testing, Yield stsrength, Elongation, Quality control, Construction materials

## Abstract

This data article describes a comprehensive dataset comprising 12,161 individual steel reinforcement bar tensile tests (3,898 test reports) collected from various construction projects across Sudan between 2016 and 2022. The data was systematically extracted from official test reports generated by the University of Khartoum, Faculty of Engineering, Department of Civil Engineering, Material and Structures Testing Laboratory. The purpose of this dataset is to establish a verified, large-scale baseline of material performance for Sudanese reinforcement steel, providing transparent and verifiable raw values of key mechanical and dimensional properties for locally sourced rebars with tested diameters ranging from 8 mm to 32 mm. This data is intended for reuse to conduct rigorous analyses on steel reinforcement quality and characteristic properties in Sudan, offering a unique baseline for regional construction quality and providing a representative performance benchmark applicable to other developing countries.

Specifications Table

**Table:** 

Subject	Engineering & Materials science
Specific subject area	*Civil Engineering – Structures and Materials - Steel Reinforcement.*
Type of data	*Raw*.
Data collection	*Data were collected by systematically extracting raw test results from official steel reinforcement tensile test reports/certificates generated at the University of Khartoum, Faculty of Engineering Material and Structures Testing Laboratory (2016–2022). Testing adhered to BS 4449 and BS EN 10080, utilizing a universal testing machine (UTM) for mechanical properties and a calibrated balance for dimensional checks. The dataset is composed of raw values as originally reported, with no preprocessing or analytical steps applied after extraction.*
Data source location	*University of Khartoum, Faculty of Engineering, Department of Civil Engineering, Material and Structures Testing Laboratory, Khartoum, Sudan.*
Data accessibility	Repository name: Mendeley Data Data identification number: https://doi.org/10.17632/hpjfmbpk9t.1 Direct URL to data: https://data.mendeley.com/datasets/hpjfmbpk9t/1
Related research article	*Large Scale Assessment of Reinforcement Steel Quality in Sudan (Submitted).*

## 1 Value of the Data

• This large-scale, raw dataset provides a verified, longitudinal baseline (2016–2022) of steel reinforcement quality in the Sudanese market, enabling academic researchers and the Sudanese Standards and Metrology Organization (SSMO) to perform statistical quality control studies to quantify production variability.
• The data enables reliability-based assessments crucial for structural safety. Structural design firms and the Sudanese Engineering Council can use raw strength distributions to perform local calibration studies for determining appropriate partial safety factors for steel.
• This resource supports national code development by providing verifiable raw elongation and dimensional data to the National Building Code Committee. It can be used to establish statistically sound local characteristic material properties, a prerequisite for defining a robust building code.
• The dataset facilitates forensic engineering and and quality monitoring for manufacturers and production engineers by offering a historical record of material properties.
• It helps forensic consultants, contractors, and the Ministry of Infrastructure trace non-compliance trends and benchmark performance within the construction sector.
• Originating from a laboratory playing a pivotal role for the SSMO, manufacturers, consultants, and contractors, this dataset provides a representative performance benchmark for a wide spectrum of stakeholders in national infrastructure projects, offering a unique baseline for regional construction quality in a developing country context.

## 2 Background

The compilation of this dataset originated from a critical need to quantify the endemic material non-conformity observed in Sudan’s construction sector. Following reports from the Sudanese Standards and Metrology Organization (SSMO) highlighting widespread use of undersized and substandard rebars, a systematic, multi-year monitoring effort was required.

The theoretical background for data generation stemmed from structural reliability engineering, which mandates that material properties used in design be statistically derived from characteristic values. The primary methodological objective was thus to create a dataset large enough for probabilistic assessments and the calibration of safety factors, which was impossible using small-sample testing.

This data article is related to an original research article analysing steel quality in Sudan. The data article adds value by decoupling the raw evidence from the interpretation. While the research article discusses the implications of variability and proposes regulatory changes, this dataset provides the raw, verifiable source values used for those analyses, allowing other researchers to validate the findings or conduct new studies on different statistical parameters or material models.

## 3 Data Description

**Fig. 1:**
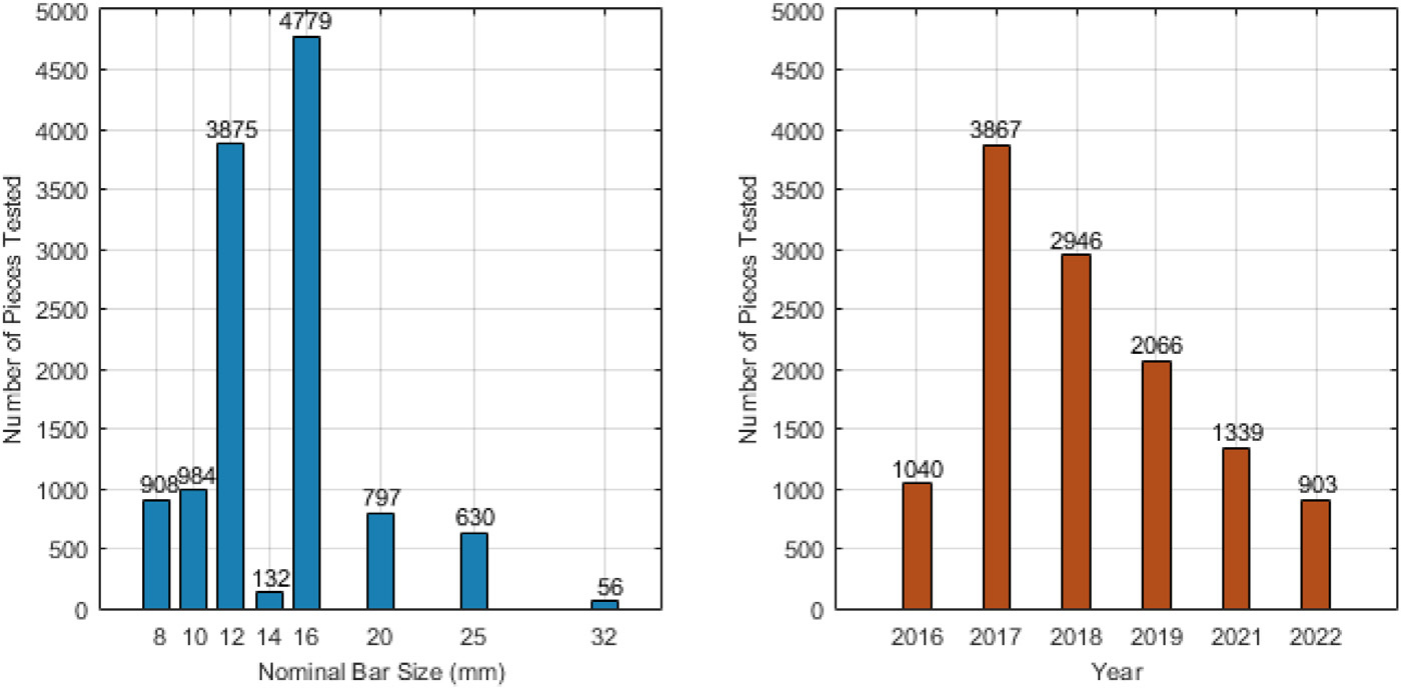
Distribution of tested rebar pieces by size (left) and year (right).

The dataset is organised in a single Microsoft Excel Workbook and deposited in Mendeley Data for easy access [1]. This workbook contains ten distinct sheets: two documentation sheets—Introduction, providing project context, and Readme, detailing column headers, standards, and unit information—and eight data sheets, one dedicated to each tested bar size: 8 mm, 10 mm, 12 mm, 14 mm, 16 mm, 20 mm, 25 mm, and 32 mm. Each of these size-specific data sheets contains the raw test results with the following uniform column headers: effective diameter (mm), diameter complies? (yes/no), Cross Sectional Area (mm^2^), Weight (kg/m), yield Strength (N/mm^2^), ultimate Strength (N/mm^2^), Elongation (%), Cold Bending Test rupture? Yes.no/not tested, and DATE. Fig. 1 presents the distribution of the tested steel rebar pieces across different bar sizes and across the years 2016 to 2022.

### 3.1 Experimental Design, Materials and Methods

The data described in this article were acquired through the systematic digitization of official steel reinforcement tensile test reports/certificates issued by the University of Khartoum, Faculty of Engineering, Department of Civil Engineering, Material and Structures Testing Laboratory over a seven-year period (2016–2022). The original tests reflected in the dataset were conducted following the specifications of BS 4449 (Steel for the reinforcement of concrete) and BS EN 10080 (Steel for the reinforcement of concrete—Weldable reinforcing steel—General) [2,3].

The data were stored in paper format (official test certificates) until this project. The digitization process was manually conducted by a dedicated team over a six-month period to ensure accuracy. Key values were transcribed into a structured electronic format using Microsoft Excel.

To ensure high data fidelity, a double-entry verification protocol was employed where digitized entries were checked against the original paper certificates. Furthermore, random spot-checks of the records were conducted to identify and correct any transcription errors.

## Limitations

The usefulness of this dataset is subject to several limitations primarily related to its acquisition and continuity, which should be carefully considered by researchers:

• Data Gaps (2020): The dataset exhibits a significant discontinuity, as test results for the year 2020 were entirely unavailable. This absence is attributed to severe operational limitations caused by the COVID-19 lockdown and concurrent political instability in Sudan, resulting in a break in the longitudinal data record.
• Loss of Historical Data: The dataset's timeline begins in 2016 because a significantly large volume of test data generated before 2016 was unfortunately destroyed during previous disruptions at the laboratory facility.
• Potential for Transcription Error: The compilation involved a six-month period of manual transcription by a dedicated team, extracting raw values from paper test certificates. Despite implementing rigorous quality checks, this manual digitization process remains a potential source of transcription errors in the final electronic dataset.
• Non-Uniform Sampling: The samples were collected based on the varying demands of construction projects and third-party submissions, not a statistically uniform plan. Consequently, the dataset reflects market demand for testing rather than a consistent, randomized sampling strategy.
• Raw stress–strain curves were not digitally archived during testing as per BS 4449/BS EN 10080 reporting standards. Yet, the dataset’s scale, comprising 12,161 tests, remains one of the largest and most unique material performance records in the region.

## Ethics Statement

The authors have read and follow the ethical requirements for publication in Data in Brief and confirming that the current work does not involve human subjects, animal experiments, or any data collected from social media platforms.

## Credit Author Statement

**Amged O. Abdelatif**: Conceptualization, Supervision, Writing – original draft. **Abdelrahim H. Abdelrahim, Gamar-Aldwla S. Shangray, Mohammed-Alfatih Mustafa, and Mustafa M. Abaker**: Data Curation. **Yahia A. Idris and Abdelrahim M. Yousif**: Testing and Data Archiving.

## Acknowledgments

The authors acknowledge the invaluable contributions of the University of Khartoum, Faculty of Engineering Material and Structures Testing Laboratory and their staff for their full support. This work is dedicated to the memory of our co-author and chief technician, Yahia A. Idris, who passed away on 4 December 2025; his dedication was instrumental to this research. This research did not receive any specific grant from funding agencies in the public, commercial, or not-for-profit sectors.

## Declaration of Competing Interest

The authors declare that they have no known competing financial interests or personal relationships that could have appeared to influence the work reported in this paper.

## Data Availability

Mendeley DataTensile Tests of Steel Reinforcement in Sudan (2016-2022) (Original data)
